# Interpretation of Health-Smart Home Data and Implications for Clinical Decision-Making: Inductive Content Analysis

**DOI:** 10.2196/75234

**Published:** 2025-11-21

**Authors:** Gordana Dermody, Diane J Cook, Roschelle L Fritz

**Affiliations:** 1 School of Health, University of the Sunshine Coast, 90 Sippy Downs Drive, Sippy Downs, 4556, Australia, 610451980220; 2 School of Electrical Engineering and Computer Science, Washington State University, Pullman, WA, United States; 3 Smart Aging and Gero Environments Lab, Betty Irene Moore School of Nursing, University of California, Davis, Sacramento, United States

**Keywords:** health-smart home, digital health, data visualization, content analysis, nursing, aging-in-place

## Abstract

**Background:**

Health-smart home technologies offer real-time sensor-based monitoring of older adult activities of daily living, allowing for early detection of changes in health. The way clinicians interpret and use this data, particularly in visualized formats, such as bar, line, and pie graphs, remains underexplored.

**Objective:**

A qualitative descriptive study design with a quantitative component was used to explore how nurses interpret sensor-derived health data from health-smart homes in 3 cases.

**Methods:**

Using an inductive content analysis approach, we analyzed nurses’ qualitative interpretations of existing sensor-derived health data from health-smart homes from 3 older adults living with ambient whole-home sensing. Nurses provided structured written feedback on visualized trends in sensor-derived health data, including activity, sleep, and mobility patterns.

**Results:**

The findings highlight both opportunities and challenges of using sensor-derived health data in older adults’ care. Nurses identified key patterns in sleep, mobility, and home engagement, but interpretation difficulties, such as unclear sleep metrics and lack of clinical context, hindered decision-making. Nurses preferred bar and line graphs over pie charts for interpreting these data. Survey results show a statistically significant difference in how nurses rated different graph types (*χ*²_2_=17.1, *P*<.001), with pie charts rated significantly lower than both bar and line graphs (*P*<.001 and *P*=.008, respectively). These findings underscore the need for improved data visualization and integration to enhance the clinical utility of sensor-derived health data from health-smart homes.

**Conclusions:**

Findings indicate that nurses were able to provide accurate interpretations of the sensor-derived health data from health-smart homes. However, there is a need for improved visualization techniques and clinician training to optimize health-smart home data for early intervention. Standardized approaches to data representation could enhance nurses’ ability to detect and act on subtle yet important information about older adults’ health changes occurring in home settings.

## Introduction

### Overview

With the global population aging and an increasing number of older adults living with chronic conditions, there is growing interest in health-smart home (HSH) technologies to support aging in place [1-3]. Traditionally, nursing homes have been the primary setting for older adult care, but concerns over the quality of care in institutional settings and a strong preference in older adults to remain living at home have led to the exploration of alternative solutions. Activities of daily living (ADLs) are routine self-care tasks, such as bathing, dressing, eating, and mobility. In the context of HSH technologies, ADLs are captured as indicators of functional health and independence. Health-smart home technologies can provide real-time, unobtrusive sensor-based monitoring of ADLs, offering opportunities for early detection of health changes [4,5]. This can help address exacerbations in chronic disease early, potentially reducing hospitalizations and unnecessary emergency department visits, and may additionally reduce costs associated with long-term residential care [6-8].

HSH technologies are particularly valuable for tracking ADLs, such as movement around the home, interest in food, toileting and hygiene, gait speed, overall activity levels, and sleep patterns [9,10]. Deviations from typical behaviors associated with these activity patterns may indicate emerging health concerns [11]. Aging in place, facilitated by HSH technologies, may not only support greater independence and social connectivity but also has the potential to reduce health care costs associated with long-term residential care. However, while HSH technologies generate large volumes of sensor-derived health data, their clinical utility depends on how well health care professionals, particularly nurses, can interpret and act on this information [11].

Despite the increasing adoption of data-driven decision-making in health care, little is known about how nurses interact with visualized HSH sensor data generated by older adults living with HSH technology, including bar, line, and pie graphs to assess trends and patterns in ADLs. Previous research has focused predominately on the technical aspects of HSH sensors, but there remains a critical gap in understanding how nurses interpret and use the HSH sensor data for decision-making [12]. The effectiveness of these technologies depends not only on the accuracy of HSH sensor data but also on how well meaningful clinical insights can be extracted from visual representations of that data [13].

This study uses a subsample dataset that includes clinicians’ interpretations of HSH sensor data that were collected for the purpose of creating a clinician-in-the-loop visual interface for nurses observing HSH sensor data derived from continuous unobtrusive sensor-based monitoring of older adults at home. Quantified findings from the larger study are published elsewhere (Ghods et al. 2018).

Distinctive to this work is our in-depth inductive qualitative analysis of narrative descriptions written by a subsample of nurses who provided clinical validation for the larger study’s clinician-in-the-loop visual analytics. In this substudy, we seek to understand how nurses think about HSH sensor data in the context of symptom presentation and illuminate challenges encountered with providing meaningful and accurate clinical interpretations. While Ghods et al [14] addressed interpretation challenges as usability barriers within a developing clinician-in-the-loop analytics system, the current substudy isolates visualization types as a key factor influencing nurses’ ability to interpret HSH sensor data. Rather than refining a single system, this substudy compares different visual formats to determine which best supports clinical reasoning, providing unique insights into how nurses cognitively process HSH sensor data.

### Purpose of the Study

The purpose of this study was to explore (1) how nurses interpret HSH sensor data derived from ambient sensors presented in bar, line, and pie graph formats and (2) to identify the challenges nurses encounter when analyzing ADL data. By applying an inductive content analysis approach [15], this study aimed to uncover key themes in nurses’ interpretations in 3 cases, assess barriers to effective data use, and provide recommendations for improving HSH sensor data visualization techniques and clinician training. The findings from this study will contribute to the development of standardized, clinically relevant HSH sensor data visualizations to enhance nurses’ ability to identify and respond to early changes in health in clients living with HSH technology.

### Background

A global trend is emerging around providing health services to older adults in their homes. It is now common for older adults to receive home care, and the sophistication of that care is advancing. In Australia, about 17% of the population is older than 65 years, and 20% receive formal in-home care or support [16]. In the European Union (EU), data from 2019 indicates that nearly 30% of people aged 65 years and older with difficulties in personal care or household activities used home care services [17]. In the United States, approximately 12 million people receive home health care services annually, with the majority being older than 65 years [18]. The United States Census Bureau estimates that by 2050 the number of persons aged 65 years and older will reach 83.9 million. Demand for home care services continues to grow. The United States Census Bureau survey (2020) found that between 2013 and 2020, usage of US home health care services increased 50.5% [19].

As older adults increasingly seek to remain living at home with the support of home health services, many face the challenge of managing chronic conditions that require ongoing monitoring and timely intervention. Conditions, such as cardiovascular disease, diabetes, respiratory disorders, and neurodegenerative diseases often contribute to functional decline, unplanned health care usage, and institutionalization in nursing homes. Despite the increasing use of home care services among older adults, many experience reduced access to primary care. For instance, a study found that older adults in the United States generally receive good quality primary care, but challenges persist in establishing and maintaining access, as well as involving patients in disease management [20].

Traditionally, older adults with higher acuity and more advanced health monitoring needs were transitioned into nursing homes where their health status could be more closely monitored. However, because costs are increasing while access is decreasing, this option is inaccessible or impractical for many [21-24]. In addition, a scoping review of barriers to accessing aged care services for culturally and linguistically diverse older adults in Australia identified issues, such as cultural insensitivity, language barriers, and affordability concerns, which further limit access to necessary services [25]. Similar barriers exist in the United States with older adults and informal caregivers affected [26]. The emergence of HSHs offers an alternative, allowing for continuous, unobtrusive monitoring of key health indicators while enabling older adults to remain safe in their own homes [2,11].

The increasing adoption of digital health tools in aging-in-place models presents new opportunities for remote patient monitoring [1,27,28]. Data visualization converts raw HSH sensor data into visually structured and easily interpretable graphics, improving comprehension, enabling trend identification, and simplifying the communication of complex information to diverse audiences.

However, while these HSH technologies generate large volumes of quantitative data, the way nurses interpret visualized HSH sensor data trends to inform decision-making remains understudied [12,18,29].

### Conceptual Perspectives

In the context of HSH technologies, poorly designed visualization interfaces—whether overly complex, visually cluttered, or misaligned with clinical needs—can obscure critical insights, delaying or even compromising patient care. Optimizing data visualization for nurses requires a balance of information density, intuitive design, and real-time interpretability to ensure digital health tools enhance, rather than hinder, decision-making and workflow efficiencies [12,30]. However, little attention has been given to the visualization of HSH sensor data. Consequently, there remains an overreliance on medical visualizations that may not align with the specific needs of nursing practice.

The medical visualization field aims to advance communication of complex medical data in a way that is understandable and actionable for health care professionals (eg, graphs and interactive visuals). This includes significant advances in human-computer interaction [29,30]. Recent advances influencing medical visualization include artificial intelligence and machine learning, the growth of wearable and ambient sensor technology, demand for telehealth and remote health monitoring, integration of augmented and virtual reality, computational power and cloud infrastructure, and personalized medicine. Examples of frequently used medical visualizations include statistical data (bar, line, and pie graphs), such as those used in patient-reported outcome measures (PROMS) and quality indicator data [31], as well as time series graphs, such as electrocardiograms (ECGs) [32], and spatial imaging (computed tomography and magnetic resonance imaging) [33]. Heatmaps and color maps are used for quickly identifying patterns in biological data collected from chemical analysis [34].

**Figure 1.**
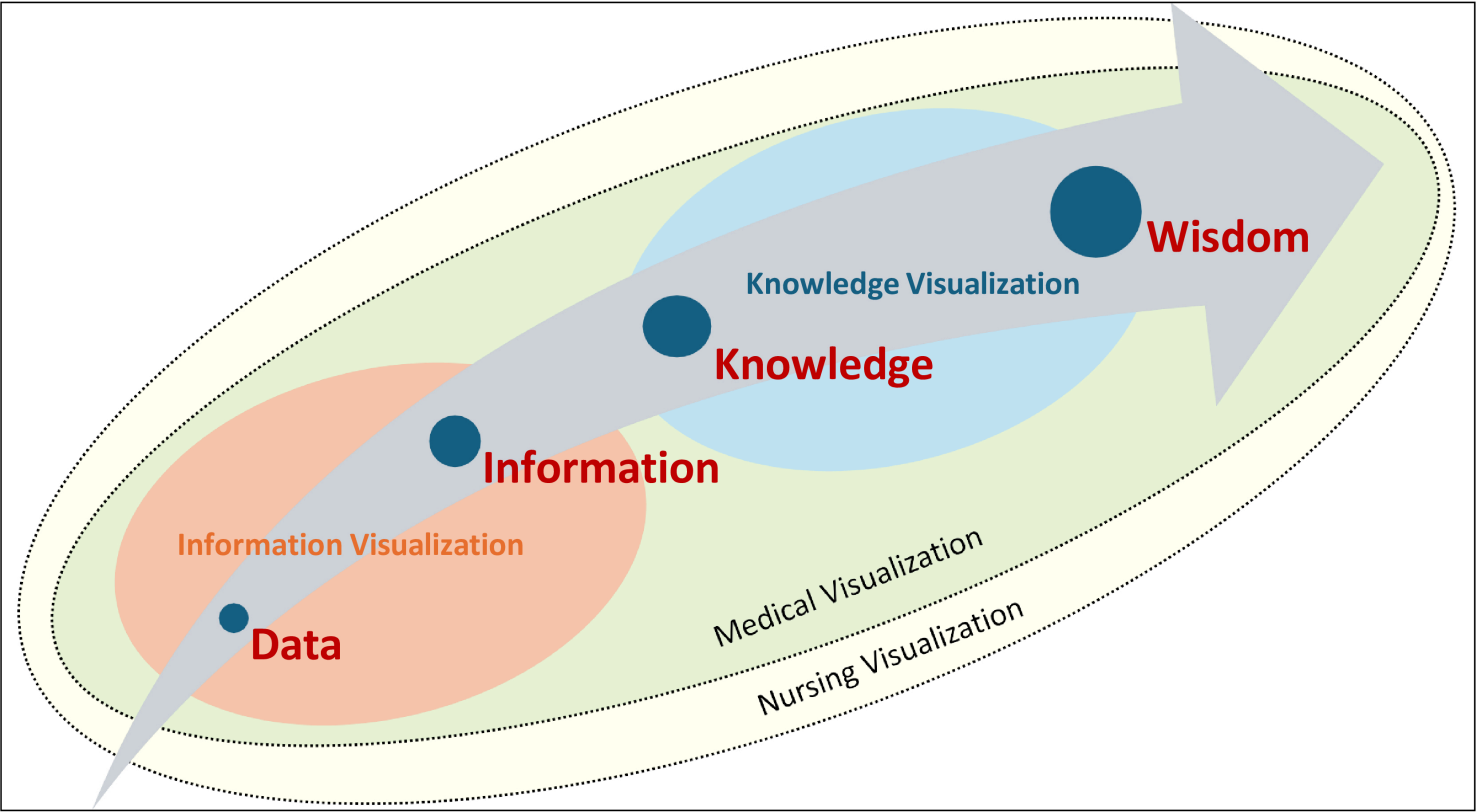
Data to wisdom continuum in the context of health-smart home data visualizations, which includes medical visualizations and much-needed nursing visualizations.

Just as statistical visualizations can mislead, suggesting false patterns or concealing key trends [35], similar risks exist in HSH sensor data. Traditional medical visualizations, such as fever charts, ECG waveforms, and blood pressure trends, focus on single physiological measures, making them intuitive for clinical interpretation. In contrast, HSH sensor data integrate multiple variables (eg, sleep, mobility, and room occupancy), requiring a more complex and contextual interpretation process. Unlike a fever chart that clearly shows temperature fluctuations, HSH sensor data visualizations must convey multiple dimensions without overwhelming users or obscuring key insights. If poorly designed, they risk misinterpretation, either overemphasizing anomalies or concealing meaningful trends. Nurses must connect disparate data points, recognize patterns, and determine clinical relevance, making the choice of visualization format critical. In addition to engaging nurses with HSH sensor data visualizations, it is important to understand how their cognitive process interprets the data. Clinical reasoning in this context requires pattern recognition, contextual knowledge, and judgment about what is clinically significant, as seen in the HSH sensor data visualizations. Effective visualizations must support, not hinder, this process, ensuring that critical health insights are easily identified and acted upon. When designing visualizations, it is important to consider the data-to-wisdom continuum and how information visualization (transforming data into useful information) and knowledge visualization (transfer of knowledge between humans) play key roles in assisting with wise clinical decision-making [12,36-39]. The data-to-wisdom model illustrates the progression of raw data to valuable, actionable insights [40]. For nurses working with HSH sensor data, the transformation from data to wisdom goes beyond medical information to include patients’ in-home situational context (ie, social and economic health determinants; Figure 1).

### Data Visualization in Nursing Decision-Making

Previous research has explored the relationship of data visualization dashboards and quality of care and clinicians’ satisfaction. Findings suggest that well-designed data visualizations improve efficiency by reducing cognitive load, enhancing pattern recognition, and supporting predictive analytics for patient care [12]. Medical visualizations are typically suited for discrete, physiologic measures (eg, temperature, ECG waveforms, and blood pressure), designed for rapid detection of deviations from a norm. By contrast, nursing visualizations often need to integrate multidimensional and contextual information (eg, ADLs, mobility, sleep, and room use), reflecting functional health and daily living patterns over time rather than isolated biomedical parameters [29,38]. While data visualization tools are well-established in acute care settings, such as adult intensive care units [12,39], their application in community-based settings such as home care remains underexplored. As health care increasingly shifts toward aging in place and remote monitoring, a growing opportunity exists to leverage HSH technologies to enhance care coordination [41]. HSHs equipped with passive sensor-based monitoring systems can provide real-time data on ADLs, mobility patterns, and other key health indicators [42]. By integrating remotely collected HSH sensor data streams into meaningful visual representations, clinicians, particularly nurses, could effectively interpret trends, identify early signs of health deterioration, intervene proactively, and work more efficiently [43]. We emphasize nurses because they are the largest group of health care professionals globally and play a central role in monitoring ADLs and functional health, particularly in community and aged care contexts where HSH technologies are most relevant. While this study refers to nurses generally, future work could examine whether visualization needs differ across contexts, such as geriatric care or assisted living. This transition from institutional to HSH sensor data visualization introduces new challenges and opportunities in designing user-friendly, clinically relevant interfaces that support remote decision-making and optimize patient outcomes [43].

### Nurses’ Interpretation of HSH Data

For decades, nurses have provided health care services to people in their homes. They collect physiological, psychosocial, and functional data that informs health care decisions [44]. Integrating remotely collected HSH sensor data of community-dwelling clients into nursing practice could enhance patient monitoring, speed up the recognition of changes in health, and promote early and proactive interventions. Studies have demonstrated that continuous HSH sensor-based monitoring in home settings enables automated detection of health events, allowing nurses to initiate preventive strategies and provide timely interventions [11]. In addition, research indicates that HSH sensor technology can detect early signs of health changes, sometimes even before patients become aware, facilitating early detection and management of health deterioration [27]. These findings support the potential of HSH technologies to augment traditional nursing assessments, leading to improved patient outcomes and supporting aging in place [11,28].

Equally critical is the design of HSH sensor data that is transformed into visualizations, which must strike a balance between simplicity and utility [38]. For nurses to leverage these tools effectively under the time pressures and cognitive demands of clinical practice, these visualizations must be intuitive and accessible, requiring minimal training to interpret, while retaining the depth and precision necessary to support meaningful interpretation and decision-making [29]. Poorly designed interfaces—whether overly complex, visually cluttered, or functionally clunky—may result in nurses not having actionable insights, posing a risk of undermining the very efficiencies and improvements that digital health tools aim to deliver [30]. Thus, understanding nurses’ interaction with HSH sensor data visualizations and optimizing design are twin imperatives for advancing care delivery in HSH technology-enabled settings.

### Research Questions

This study aimed to explore:

1. How nurses interpret HSH sensor data visualizations and the clinical implications of their interpretations.
2. How nurses interpret bar, line, and pie graphs displaying HSH sensor data?
3. What patterns and challenges emerge in their analysis of patient activity, sleep, and mobility?

## Methods

### Study Design

This study used a qualitative descriptive design with inductive content analysis, an approach well-suited for exploring participants’ experiences, perceptions, and insights [15,45]. Qualitative descriptive research focuses on exploring participants’ experiences and perspectives, staying close to their words and meanings without extensive interpretation or theoretical abstraction [46], making it particularly valuable in health care research where understanding practical applications and real-world experiences is essential.

### Sample and Recruitment

This study used a combination of purposive and convenience sampling to recruit participants with relevant expertise in nursing practice, health informatics, and education. Purposive sampling was used to ensure the inclusion of Registered Nurses (RNs) with at least 5 years or more experience working in clinical practice, clinical decision-making, and exposure to health informatics.

Convenience sampling was applied due to the accessibility of participants within academic and professional networks. Recruitment was conducted via course announcements, professional networks, and targeted email invitations. Study participants (N=14) were experienced RNs who were either (1) enrolled in a Doctor of Nursing Practice Family Nurse Practitioner program at a university in the Pacific Northwest region of the United States and were completing a graduate course in health informatics or (2) nursing faculty from various schools of nursing (associate, bachelor’s, or graduate nursing degree programs) in the Pacific Northwest region of the United States. All participants held a bachelor’s or higher nursing degree from accredited nursing schools in the United States, and all were female. Age was not obtained. The sample for this substudy is a subset of a total RN sample of (N=60) described elsewhere (Ghods et al [14]). The nursing text-based data analyzed here were provided by the nurses (N=14) who provided clinical validation for the larger study.

### Research Team and Reflexivity

All researchers (RLF, DJC, and GD) held academic positions in research-intensive universities. DJC held an academic position in engineering; RLF and GD taught in various nursing programs All reserachers are females with PhDs. RLF and GD are RNs with extensive clinical nursing experience spanning several decades. All have have expertise in deploying health-smart homes and conducting qualitative and mixed research. In addition, they have personal experience caring for frail older family members, including those with dementia, which informs their vested interest in advancing the integration of health-smart homes into new models of care. RLF introduced the study and full study purpose to the potential participants. Participants were not interviewed; instead, they provided written responses.

### Data Collection Approach

**Figure 2.**
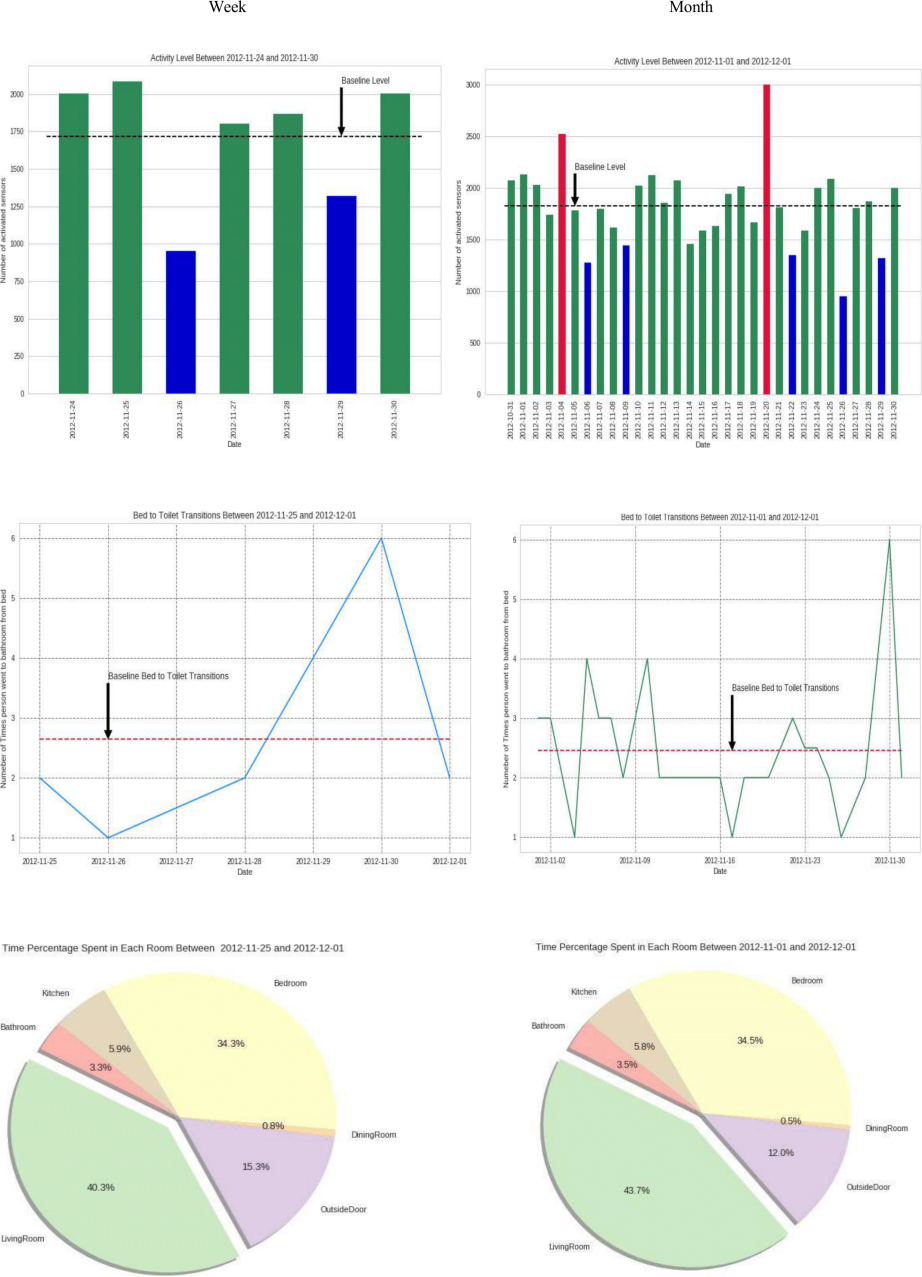
Exemplar health-smart home sensor data visualizations that Registered Nurse participants were asked to interpret.

Nurses who consented to the study were sent an asynchronous email with a Microsoft PowerPoint document (without audio-video) with a 5-slide, written explanation of the health-smart home as well as a short instruction manual on how to use the interactive data visualization tool prepared using Grafana, which was accessible using a web link. All study activities were completed in the private time of the participants. The Microsoft PowerPoint presentation introduced 3 participant cases, each with background information, including biological sex, age, primary diagnosis, and a specified 7-day period for analysis. The cases were purposively selected to represent variation in age and diagnosis among older adults living alone, which was consistent with the study aim of exploring how nurses interpret HSH sensor data relevant to community-dwelling older adults: (1) Case 1 was an 87-year-old female with lung cancer undergoing daily radiation treatments, (2) Case 2 was a 92-year-old female with nonspecific insomnia, and (3) Case 3 was an 89-year-old female with no medical history provided. Nurses were provided with a home floor plan with sensor placements and multiple Grafana-generated HSH sensor data visualizations, including bar graphs for activity levels and sleep duration (weekly and monthly), line graphs for sleep interruptions and walking speed (weekly and monthly), and pie charts representing time spent in different home locations (weekly and monthly). While 31-day visualizations were provided for context, the health event of interest occurred within a specified 7-day window, which was the primary focus of analysis and purposefully selected by the researchers. Nurses could draw on either the 7-day or 31-day visualizations when making their interpretations, but the health event of interest was located within the defined 7-day window. The HSH sensor data used in the visualizations for this study were from the HSH sensors placed in the homes of community-dwelling older adult participants from multiple health-smart home studies conducted at Washington State University’s Center for Advanced Studies in Adaptive Systems [47]. An example of the visualizations provided to participants is shown in Figure 2. Nurses accessed the Grafana Dashboard to review these HSH sensor data visualizations and respond to structured questions based on the specific data window required.

HSH sensor data included passive infrared motion, temperature, humidity, light, and door use (magnetic) contact sensors placed on the ceilings and walls of older adults’ residences. To facilitate data exploration and pattern identification, Grafana was used to generate a dashboard with interactive visualizations of the HSH sensor data. The details on how the visualizations were developed are described elsewhere [14]. Grafana is an open-source data visualization and monitoring platform that integrates with various data sources, including Prometheus (a monitoring system for collecting and querying time-series data), InfluxDB (a high-performance database designed for time-stamped HSH sensor data), and MySQL (a widely used relational database for structured data storage). These integrations enable Grafana to aggregate and display real-time and historical health-related data, making it a valuable tool for nursing research, digital health applications, and HSH monitoring.

Participants responded to a combination of 9 structured questions (1 open-ended) and a 7-point Likert scale survey, adapted specifically for this study [48], which was not pilot tested. The structured questions (Textbox 1) aimed to assess nurses’ interpretations of the visualized data and their perceptions of its usability. Participants were asked to identify any changes in health status or events, describe their clinical interpretations based on the graphs, and specify which charts they used in their decision-making. Additional questions explored graph interpretability, including which visual formats were easiest to use, how the graphs could be improved, and whether alternative graph types would better represent specific data points (eg, activity level, sleep duration, and walking speed). Participants also provided general feedback on the overall usefulness of the visualizations, indicated whether any relevant information was missing or unnecessary, and offered suggestions for redesigning the graphs (eg, modifications to colors, axis scales, or graph types). Finally, participants recorded the approximate time spent on the tasks, providing insight into the cognitive workload associated with interpreting the data.

Textbox 1. **Structured questions**

- What change in health status or health event (if any) do you identify for this patient?
- What clinical interpretations would you make about this patient based on these graphs?
- Which charts did you use to make your interpretations?
- Which types of graphs were easiest to interpret? How could these graphs be improved to make them easier and more efficient to interpret?
- Would it be more useful to see any of the characteristics (ie, activity level, sleep duration, sleep interruptions, walking speed, and percentage of time spent in locations) represented by a different type of graph?
- Please comment on the overall usefulness of the graphs.
- Is there any information you would like to see on the visualization that is not present? Is there any existing information on the visualization that was not useful to you?
- Please provide any comments or suggestions you have about how you would redesign this visualization (eg, colors, type of graph or chart, scales of x and y axes).
- Please record the approximate amount of time you spent working on these tasks.

### Survey

The Post-Study System Usability Questionnaire (PSSUQ), developed by Lewis [48], is an 18-item instrument designed to evaluate user satisfaction with system usability, structured around 3 factors, namely System Usefulness, Information Quality, and Interface Quality. The scale uses a 7-point Likert format (1=strongly agree and 7=strongly disagree), with high internal reliability (Cronbach α=0.97).

For this study, an adapted version of the PSSUQ was used, comprising 11 selected items tailored to assess nurses’ perceptions of smart home data visualizations. The modifications ensured relevance to clinical usability, ease of interpretation, and decision-making support while maintaining the original scale’s structure and reliability. The survey was used to gain preliminary insights into the participants’ views on the usability and clinical relevance of the different visualization types, including bar, line, and pie graphs (Table 1). The selected 11 items align with established principles of usability assessment, particularly in the context of health informatics and clinical decision support tools. These questions assessed key domains of visualization effectiveness, ensuring that the presented data support accurate interpretation and informed clinical decision-making. Participants were instructed to rate each statement for each visualization graph type (bar, line, and pie) on a 7-point Likert scale, with response options of 1 (strongly agree), 2 (agree), 3 (somewhat agree), 4 (neutral), 5 (somewhat disagree), 6 (disagree), and 7 (strongly disagree). A numbered scale from 1=strongly agree to 7=strongly disagree was displayed at the top of the questionnaire to ensure clarity in response options. In selecting a 7-point Likert scale over a 5-point scale, the goal was to enhance response sensitivity and capture more nuanced participant perceptions. However, the primary goal of this study was not to generalize statistical findings, but rather to provide contextual and quantifiable information to gain additional insights into the qualitative exploration of nurses’ interpretations of the HSH sensor data visualizations. The idea was that the Likert-scale responses would offer structured insights into the qualitative perceptions of usability and data clarity while maintaining the exploratory nature of this research.

**Table 1.** Health-smart home survey.

Interpretation	Meaning
Comprehensibility and ease of use	
“Overall, I am satisfied with how easy it is to understand the visualization of the data.” [P1] “The information provided by the visualization was easy to understand.” [P6] “The organization of the visual data was clear.” [P7]	These items assess cognitive load and how intuitively nurses can interpret the data without additional explanation, an essential factor in clinical decision support.
Interpretation and clinical meaning	
“I was able to efficiently interpret the visual data.” [P2] “The visual data is presented in a clinically meaningful way.” [P9] “I am confident that visualizing data in this format would allow me to identify clinically meaningful change in health status.” [P10]	These items measure the effectiveness of the visualization format in enabling clinicians to recognize trends, patterns, and health changes in a way that informs practice.
Usability and workflow integration	
“I felt comfortable interpreting this visualization.” [P3] “I believe I could become productive quickly using this visualization.” [P4] “It was easy to find the information I needed in this visualization.” [P5]	These statements reflect usability heuristics, such as learnability, efficiency, and confidence in decision-making, which are critical for technology adoption in nursing.
Data completeness and satisfaction	
“The visualization contains all of the information I would expect it to have.” [P8] “Overall, I am satisfied with this visualization.” [P11]	These items assess perceived completeness and user satisfaction, which influence engagement and continued use of visualization tools in clinical settings.

### Data Analysis Approach

#### Text-Based Evaluation Data

This study used general inductive content analysis [15] to analyze nurses’ written feedback on structured questions related to HSH sensor data visualizations, including graphs depicting sleep, activity, and mobility trends of 3 smart home participant cases. Content analysis is a systematic and objective research method used to describe and quantify phenomena, often applied to document analysis. It enables researchers to organize data into meaningful categories, assuming that words and phrases classified together share a common meaning [15,49]. This approach helps test theoretical concepts, derive insights, and generate practical knowledge [50]. By applying an inductive content analysis approach, this study aimed to uncover key themes in nurses’ interpretations, assess barriers to effective data use, and provide recommendations for improving data visualization techniques and clinician training. The goal of the researchers was to capture the breadth and depth of participant feedback to ensure a comprehensive understanding of the data. Accordingly, for this study, data saturation was not the intended criterion for determining adequacy of data; rather, the approach focused on ensuring exploration of participant perspectives and identifying patterns and variations in responses. Therefore, the analysis aimed to reflect the full range of participant experiences rather than achieving theoretical saturation. Data were analyzed by GD and RLF following a 3-phase inductive process described by Thomas [15]. In Phase 1 (open coding), 2 researchers (GD and RLF) independently reviewed participants’ responses, identifying key statements and applying initial descriptive codes. In Phase 2 (categorization), similar codes were grouped by GD and RLF into broader categories to reflect patterns in how nurses engaged with the visualizations. In Phase 3 (theme development), categories were synthesized (GD and RLF) into higher-order themes, representing overarching interpretations of how the visual data were perceived and used. Throughout the process, the researchers engaged in reflexive discussions to ensure that the themes emerged from the data rather than being imposed. To enhance trustworthiness and rigor, both researchers independently coded the data and then met to compare interpretations, resolving discrepancies through discussion. Manual coding was used by GD and RLF to maintain close engagement with the data, rather than relying on qualitative analysis software. Representative participant quotations were selected to illustrate each theme, ensuring transparency in how interpretations were derived from the raw data. Participants were not asked to provide feedback on the findings.

### Survey

Descriptive statistics were computed using SPSS (version 30; IBM Corp) to summarize participants’ perceptions of the 3 visualization types (bar, line, and pie). Mean and SD values were calculated for each of the 11 survey items across the 3 visualization types. Given the Likert scale structure (1=strongly agree and 7=strongly disagree), lower mean scores indicate stronger agreement with a statement, while higher mean scores indicate greater disagreement. These summary statistics provided an initial assessment of perceived usability, interpretability, and clinical relevance across the 3 visualization types. Since participants responded to the same set of 11 survey items for all 3 visualization types, the dataset was treated as repeated measures, allowing for within-subject comparisons. A Friedman test was conducted to determine whether there were significant differences in ratings across the 3 visualization types. The Friedman test is a nonparametric alternative to repeated-measures ANOVA, appropriate for ordinal Likert-scale data and small sample sizes (N=14). To ensure that within-subject variability was preserved, each individual response across all 11 survey items was included in the analysis rather than averaging responses at the participant level.

If a significant difference was detected, post hoc pairwise comparisons were conducted using the Wilcoxon signed-rank test, a nonparametric test for paired samples, to identify specific differences between graph types (bar vs line, bar vs pie, and line vs pie). An alpha level of .05 was used to determine statistical significance for all analyses [51].

### Ethical Considerations

This study was determined to be exempt research by the Washington State University Office of Research Assurances (Institutional Review Board number 16457). Participation was voluntary, with informed consent, and participants could withdraw at any time before submitting their responses, which were treated privately and confidentially, ensuring autonomy and adherence to ethical research principles. For those enrolled in courses, participation had no bearing on grades or academic standing, ensuring that responses were given freely and without obligation. After participants submitted their responses, all written responses were deidentified, and the associated email was deleted, making it impossible to retract or exclude a response after submission. Compensation was not offered.

## Results

### Text-Based Evaluation Data

All RNs (N=14) responded to the structured questions for each of the 3 smart home participants and completed the 11-question Likert scale survey. No participants withdrew. All participants answered all structured qualitative questions in writing across the 3 smart home participant cases and completed the 11 survey questions for bar, line, and pie visualizations. The average time reported by participants spent working on reviewing the health-smart home data visualization across all 3 smart home participant cases and providing written responses was 71 minutes.

### Inductive Content Analysis of Nurses’ Evaluations

#### Case 1

The qualitative analysis of nurses’ evaluations of HSH sensor data visualizations for smart home participants Case 1, an 87-year-old female living on her own with a diagnosis of lung cancer undergoing radiation treatments 5 days per week, revealed four overarching themes: (1) sleep disruptions and nocturnal activity, (2) mobility declines and fatigue-related activity fluctuations, (3) home environment usage and social engagement, and (4) challenges in interpreting smart home data. These themes reflect how nurses interpreted the patient’s sleep, mobility, and activity patterns, as well as the challenges they encountered in using HSH sensor data visualizations for clinical decision-making.

##### Sleep Disruptions and Nocturnal Activity

Nurses identified significant sleep disturbances in smart home participant Case 1, particularly in relation to the patient’s radiation treatment cycle. HSH sensor data visualizations showed erratic sleep patterns, with poor sleep the night before treatment and longer rest posttreatment, which nurses linked to fatigue and recovery. In addition, frequent nocturnal awakenings and bed-to-toilet transitions were observed, leading nurses to speculate on possible urinary issues, discomfort, or pain-related sleep disruption. One nurse noted, *“*Sleep is being interrupted,” (P12) while another highlighted the increasing bed-to-toilet transitions, stating:

*She had less transitions from bed-to-toilet after her radiation and then slowly increased in frequency over the week.*

[P4]

One participant noted that more information is needed to make clinically relevant inferences, noting:

*Only looking at night-time use of bathroom does not give me a good enough clinical picture. Was there something that occurred during the before the nighttime of increased bathroom use that would explain it? (increased consumption of liquids, change in meds?) To many unknowns to draw meaning here.*

[P14]

In addition, a recurring challenge was distinguishing true sleep from inactivity, as nurses noted uncertainty in interpreting whether long sleep durations indicated restful sleep or prolonged immobility due to illness or fatigue. One participant expressed this challenge, stating:

*It is hard to tell if she is getting up or just rolling around.*

[P2]

Some nurses suggested anxiety before treatment may have contributed to pretreatment sleep loss, as one remarked:

*The patient slept very little and poorly the night before radiation treatments.*

[P5]

###### Clinical Implications

Nurses linked sleep disturbances in smart home participant Case 1 to health status and treatment effects, emphasizing the need for improved sleep monitoring methods.

They suggested potential interventions to address nocturia and anxiety-related sleep loss, highlighting the importance of assessing sleep in conjunction with other clinical indicators. However, one participant noted that more information was needed to make clinical inferences:

*Patient with more sleep interruptions last ½ of month. This could be clinically relevant, depending on the diagnosis and medications/doses (or changes in meds/doses). Need more context. As is, cannot infer anything.*

[P14]

### Mobility Decline and Fatigue-Related Activity Fluctuations

HSH sensor data visualizations for smart home participant Case 1 indicated progressive mobility decline, which nurses interpreted as radiation-related fatigue. A noticeable reduction in walking speed was observed throughout the treatment cycle, with the slowest movement occurring on the fourth and fifth treatment days. One nurse noted:

*Thursday and Friday (4‐5 treatments of Radiation), the patient’s walking speed slows due to fatigue, pain, and/or discomfort and the patient takes a significantly longer time getting to the bathroom at day 4 or 5 of radiation.*

[P5]

Another participant observed:

*The participant’s walking speed declined progressively throughout the treatment week, with the slowest movement observed on the fourth and fifth treatment days.*

[P3]

Nurses also noted increased sedentary behavior, as smart home participant Case 1 spent more time with “less activity on the third and sixth days of this week” (P10)*,* or sitting post treatment, which they attributed to fatigue, pain, and overall physical decline. One participant described this as:

*The patient spends a lot of time watching TV and being sedentary in the living room.*

[P5]

In addition, delayed and more difficult bathroom transitions were observed, leading some nurses to express concern about increased fall risk, particularly toward the end of the treatment week. One nurse warned:

*This patient could experience an increased risk for falls due to the number of bed transfers.*

[P4]

#### Clinical Implications

Nurses emphasized the need for closer monitoring of mobility patterns in smart home participant Case 1, particularly in relation to fall risk and functional decline. They suggested that gait assessments and tracking bathroom transitions over time could provide early warning signs of worsening fatigue and mobility impairments.

### Home Environment Usage and Social Engagement

HSH sensor data visualizations for smart home participant Case 1 provided insights into daily routines and home environment use. Nurses observed consistent room usage, with most time spent in the bedroom and living room, while minimal time was spent in the kitchen and dining areas. One participant noted:

*Minimal time in the bathroom and kitchen. Equal time in living room and bedroom. Time is spent outside.*

[P1]

This led to speculation about possible reduced meal preparation and food intake, suggesting potential nutritional concerns. Limited outdoor activity was also noted, with smart home participant Case 1 leaving home primarily for medical appointments. Some nurses speculated that frequent door openings could indicate smoking habits or social visits, though this remained unclear. One participant questioned:

*There were periods of time where the door would open and close and then open and close again minutes later throughout the day, I wondered if possibly the patient was going outside to smoke but that could just be my bias.*

[P11]

#### Clinical Implications

Nurses highlighted fatigue-related lifestyle changes that may require nutritional support and strategies to promote social engagement for smart home participant Case 1. Understanding patterns of home and community interaction may provide useful indicators of overall well-being and functional status.

### Challenges in Interpreting Smart Home Data

Despite the valuable insights provided by the HSH sensor data visualizations, nurses faced multiple challenges in interpretation for smart home participant, Case 1. Walking speed metrics were particularly difficult to assess, as nurses questioned whether changes reflected fatigue, pain, or another condition. One participant expressed confusion, stating:

*I do not know how to interpret walking speed. What is the unit of measure? Does walking slower mean the patient was in pain? More sleepy? Less alert?*

[P6]

Sleep data interpretation was another challenge, with nurses struggling to differentiate between sleep and prolonged inactivity and lacking more context, making it harder to determine true sleep quality.

*Is the baseline specific to the patient? Or is it a standardized baseline? I wonder why the slow increase in sleep hours in the week?*

[P13]

Another participant noted:

*On the previous graph, 11/30 showed that the patient slept more hours than usual. I think perhaps what the graph was showing was how many hours they were in bed and not how many hours they were sleeping as this graph shows the patient was up 6 times to go to the bathroom.*

[P6]

One participant remarked:

*Patient with more sleep interruptions last ½ of month. This could be clinically relevant, depending on the diagnosis and medications/doses (or changes in meds/doses). Need more context. As is, cannot infer anything.*

[P14]

Regarding data visualization, nurses found that line graphs and trend analyses were more useful, whereas pie charts were seen as less informative. One participant stated:

*I used the graph charts and the links to the sensor data. The pie chart didn’t provide much information.*

[P1]

In addition, nurses noted that raw sensor data alone lacked sufficient clinical context, reinforcing the need for integrating HSH sensor data with clinical benchmarks to enhance usability.

*Not sure what was happening on 11/26, was the patient not home? I could look at the raw sensor data, but not sure if that would answer the question.*

[P13]

#### Clinical Implications

Nurses emphasized the need for enhanced data visualization techniques, including clearer reference values for mobility metrics and improved methods for interpreting sleep data for smart home participant Case 1. They also highlighted the importance of pairing HSH sensor data with clinical evaluations to ensure that findings are clinically meaningful and actionable. In summary, nurses effectively used HSH sensor data to identify key patterns related to sleep, mobility, and home activity in smart home participant Case 1, linking these trends to radiation therapy effects, fatigue, and potential health risks. However, challenges in data interpretation highlighted the need for improved contextualization, enhanced data visualization, and clearer clinical benchmarks to optimize the integration of smart home technology into patient care.

### Case 2

The qualitative analysis of nurses’ evaluations of HSH sensor data visualizations for smart home participants Case 2, a 92-year-old female living alone with a diagnosis of nonspecific insomnia, revealed four overarching themes: (1) chronic sleep disruptions and nighttime activity, (2) nighttime bathroom usage and health implications, (3) activity patterns: stability with periodic declines, and (4) low engagement and reduced activity levels. These themes illustrate nurses’ interpretations of the patient’s sleep, mobility, and home environment usage while highlighting the challenges they encountered in making clinical assessments using HSH sensor data visualizations.

#### Chronic Sleep Disruptions and Nighttime Activity

Nurses identified severe sleep disturbances in smart home participant Case 2, with erratic sleep patterns making it difficult to establish a consistent sleep schedule. HSH sensor data visualizations revealed alternating periods of no sleep and oversleeping, suggesting disordered sleep cycles.

Frequent nocturnal awakenings were also common, with the participant waking between 2 and 8 times per night. One nurse noted:

*On average there were 5 hours of sleep and a few times there were 9 hours or below 2 hours.*

[P1]

Another participant highlighted the frequency of awakenings, stating*:*

*Frequently disturbed sleep, with some periods of being considerably disrupted.*

[P2]

Poor sleep hygiene was also a concern, with some nurses speculating that bedroom use patterns indicated nonsleep activities, such as watching television. In addition, nurses noted inconsistencies in HSH sensor data visualizations, with one nurse commenting:

*She does not appear to have a decrease in sensor activity that would allow me to interpret her amount of sleep.*

[P4]

This raised concerns about whether HSH sensor data visualizations could reliably differentiate true sleep from stillness or inactivity. The potential relationship between different patterns, such as insomnia and slower walking, was noted:

*Most of the month she is moving slower than her norm. Not sure this has anything to do with insomnia, or maybe insomnia is only one of the factors affecting walking speed.*

[P14]

##### Clinical Implications

Nurses recommended improved sleep monitoring techniques to better distinguish sleep from prolonged stillness. In addition, they suggested potential sleep hygiene interventions to optimize conditions for sleep and reduce nighttime awakenings. Given the frequent disruptions, some nurses also proposed further evaluation for nocturia or an overactive bladder as a possible contributor to fragmented sleep.

### Nighttime Bathroom Usage and Health Implications

A consistent finding in smart home participant Case 2 was the impact of frequent nighttime bathroom trips on sleep quality. The participant exhibited high rates of nighttime bed-to-toilet transitions, with increased bathroom visits before and after sleepless nights. One nurse observed:

*Her bed-to-toilet transitions are above her baseline over 50% of the time.*

[P4]

Another nurse noted:

*Patient’s sleep interruptions varied from being below her baseline half of the month and spikes in the middle and end of the month showing she was interrupted more frequently to go to the bathroom (6‐8 times) ‘Why is the baseline different for the week and the month?’*

[P8]

Some nurses speculated that the participant may have undiagnosed urinary issues, such as nocturia or an overactive bladder, which exacerbated insomnia. One nurse suggested:

*She has an overactive bladder and needs evaluation to rule out other possibilities related to her urinary health or dietary plan (too much caffeine).*

[P12]

Fluctuations in bathroom visit frequency were also noted, with spikes occurring at certain times of the month, raising questions about whether this pattern correlated with medications, sleeplessness, hydration habits, or other health factors.

*More trips to the bathroom occur when the patient is not sleeping well, or does the patient not sleep well because always getting up to the bathroom?*

[P13]

#### Clinical Implications

Nurses recommended further clinical evaluation for nocturia or overactive bladder, given its potential role in disrupted sleep patterns. They also suggested tracking fluid intake, medication use, and other behavioral factors to assess whether they contributed to nighttime awakenings. However, additional clinical correlation was deemed necessary to determine whether nocturia was a primary cause of sleep disturbances or a secondary effect of another condition.

### Activity Patterns: Stability with Periodic Declines

The overall activity levels for Patient Case 2 remained relatively stable, but nurses observed fatigue-related fluctuations that affected mobility. The patient’s baseline activity level was generally consistent, but some days exhibited extreme drops. One nurse noted:

*The patient was close to her baseline activity most of the month with 4 days of increased activity. She had 7 days of the month where she was below her activity baseline.*

[P7]

Fatigue-related declines in mobility were particularly evident after nights of poor sleep, leading nurses to speculate that sleep deprivation contributed to daytime inactivity. One participant stated:

*Walking speed decreased for about one week out of the month.*

[P2]

However, occasional spikes in activity were also noted, leading some nurses to question whether these fluctuations were linked to medication effects, visitors, or anxiety-related restlessness. One nurse commented:

*This patient had a huge increase in their relative walking speed the week in focus compared to the entire month.*

[P4]

#### Clinical Implications

Nurses emphasized the need for regular mobility assessments to monitor gait stability and assess fall risk. Some nurses suggested that walking speed data required clearer reference points to determine whether changes were clinically significant or within normal variability. Others proposed that increased mobility episodes could be linked to anxiety or restlessness, warranting further evaluation.

### Low Engagement and Reduced Activity Levels

HSH sensor data visualizations revealed patterns of low engagement and limited activity within the home environment. Nurses observed that Patient Case 2 spent most of the time in the bedroom and living room, with minimal time in the kitchen or other areas. One nurse noted:

*Majority of time is in bedroom, rarely outside. Not using work area (not sure what this is). Little time in kitchen but could be prepping food and eating in another room.*

[P1]

Another participant speculated that this pattern reflected social withdrawal or reduced motivation for daily activities

*Patient spends the majority of her time in her bedroom and living room. She spends very little time in the other rooms. She is in the bathroom more than she is in the kitchen and dining room. She doesn’t go outside or lets in visitors much.*

[P7]

While activity levels remained mostly low and stable, occasional short bursts of movement were observed. Some nurses speculated that these brief periods of increased movement might be linked to anxiety, discomfort, or external factors.

#### Clinical Implications

Nurses recommended further assessment of the patient’s social and emotional well-being to determine whether isolation, depression, or other mental health factors contributed to low engagement. In addition, some suggested that light physical activity or a structured daily routine might help reduce prolonged sedentary behavior. Given the minimal kitchen use, nurses also recommended tracking mealtime routines to assess for dietary concerns.

In summary, nurses interpreted HSH sensor data visualizations from smart home participant Case 2 to identify chronic sleep disruptions, frequent nighttime awakenings, fluctuations in mobility, and patterns of low engagement. While the HSH sensor data visualizations provided valuable insights into daily routines and health behaviors, nurses faced challenges in data interpretation, particularly in distinguishing true sleep from stillness, assessing the clinical significance of mobility changes, and linking nocturnal bathroom usage to underlying conditions. These findings highlight the need for improved contextualization, enhanced data visualization techniques, and clearer clinical reference points to optimize the integration of smart home technology into patient care.

### Case 3

The qualitative analysis of nurses’ evaluations of HSH sensor data visualizations for smart home participants Case 3, an 89-year-old female living alone without a medical history provided, revealed five overarching themes: (1) fluctuating sleep and activity patterns, (2) increased nighttime bathroom trips and their impact on sleep, (3) walking speed decline and mobility variability, (4) home usage patterns and sedentary lifestyle, and (5) challenges in data interpretation and clinical context. These findings reflect nurses’ interpretations of the patient’s sleep, mobility, and home environment usage, as well as the limitations they faced in drawing clinical insights due to missing medical history.

#### Fluctuating Sleep and Activity Patterns

Nurses observed erratic sleep behaviors and inconsistent activity levels, making it difficult to establish a stable daily routine for Case 3. The HSH sensor data visualizations indicated persistent low sleep levels, with frequent awakenings affecting rest. Sleep duration ranged from as little as 3 hours to over 7 hours, with no discernible pattern. One nurse noted, “Average 5‐7 hours’ sleep. Few greater and one 3 hours” (P1)*.* In addition, nighttime restlessness was evident, as HSH sensor activity remained high even during supposed sleep periods. One nurse noted, *“*great variety in sleep with more fluctuation towards the end of the month” (P6). Another nurse noted an interesting pattern of insomnia and then sleep recovery, stating:

*Patient is having some nights where she doesn’t sleep much but tends to follow this with a good sleep day (recovery).*

[P14]

There were concerns about whether HSH sensor-detected inactivity truly reflected sleep.

*Over the month, sleep interruptions hovered close to baseline. There was one spike mid-month where her sleep interruptions increased dramatically to 20 times.*

[P7]

Furthermore, activity levels varied significantly, with some days showing increased movement followed by sharp declines, making overall behavioral patterns difficult to interpret.

*I am unable to make any interpretations based off of this information.*

[P4]

##### Clinical Implications

Nurses suggested that erratic sleep cycles could contribute to fatigue, cognitive impairments, and reduced mobility. They recommended further assessment of sleep hygiene and potential interventions for insomnia or anxiety. In addition, tracking factors such as caffeine intake, medication effects, or psychological stressors could help clarify the underlying causes of sleep disturbances.

### Increased Nighttime Bathroom Trips and Their Impact on Sleep

A key finding in nurses’ interpretations was the high frequency of nighttime bed-to-toilet transitions, which appeared to correlate with poor sleep quality. Smart home participant Case 3 woke up multiple times per night, with some nights exceeding 20 trips to the bathroom. One nurse stated:

*The patient barely slept on 3 of 7 days and 4 other days patient slept 7 to 9 hours.*

[P3]

Another participant noted:

*The patients transfers [to toilet] increased over 50% of their baseline during the week in observation. With days 10/03/17 & 10/07/17 showing less than baseline activity.*

[P4]

Nurses speculated that the frequent nocturnal awakenings could indicate undiagnosed urinary health issues, such as nocturia, an overactive bladder, or a possible urinary tract infection. One nurse commented:

*Possible UTI or increased diuresis? Patient awakens in spikes to use the bathroom at night.*

[P6]

In addition, nurses noted a clear correlation between bathroom use and sleep disruptions, where nights with fewer bathroom trips were associated with longer and more restful sleep.

#### Clinical Implications

Nurses recommended further assessment of urinary function, hydration patterns, and medication side effects to determine if nocturia was a primary cause of sleep disturbances. They also suggested dietary adjustments (eg, limiting caffeine intake) and nighttime toileting strategies to help mitigate sleep disruptions.

### Walking Speed Decline and Mobility Variability

Nurses examined walking speed data to assess mobility and potential health risks, identifying notable trends for smart home participant Case 3. The participant exhibited significant fluctuations in walking speed, with some days showing markedly slow movement followed by brief spikes in speed. One nurse noted, “Walking speed fluctuated greatly throughout the week” (P6*).* There was a clear correlation between bathroom trips and walking speed, with nurses observing that on nights with fewer bathroom trips, walking speed was faster, whereas frequent nighttime awakenings led to slower movement. One nurse stated:

*The patient walks more quickly on nights that she gets more sleep. This could be due to urgency.*

[P11]

Another participant added:

*The patient walks quickly on the nights that she goes to the bathroom less.*

[P5]

In addition, a gradual decline in mobility over time was noted, with nurses raising concerns about fatigue, potential muscle weakness, or an undiagnosed condition. One nurse commented, “Significant decreased walking speed overall” (P1*).*

#### Clinical Implications

Nurses highlighted that variability in walking speed could indicate episodic changes in energy levels, urgency, or discomfort. They recommended tracking patterns between sleep, fatigue, and mobility changes to provide insights into the patient’s overall health status. Further assessment for fall risk or muscle weakness was suggested to identify early mobility decline.

### Home Usage Patterns and Sedentary Lifestyle

HSH sensor data visualizations provided insights into smart home participant Case 3’s daily movement within her home, revealing low engagement and limited room usage. The participant spent most of her time in the bedroom and living room, with minimal movement to the kitchen, dining room, or office. One nurse noted:

Another participant added, “Majority of time is in bedroom, rarely outside” (P3)*.* Nurses speculated that limited home engagement could be a sign of social withdrawal, cognitive decline, or fatigue-related lifestyle changes. One participant remarked:

*he patient spent less time in the office and more time in the bathroom during the week where she had two nights of poor sleep. She also spent much less time in the kitchen and less time outside.*

[P5]

In addition, some nurses observed that periods of poor sleep or increased bathroom trips correlated with reduced movement throughout the home during the day, further reinforcing the connection between nighttime disruptions and daytime fatigue.

#### Clinical Implications

Nurses suggested encouraging structured movement, social interaction, or small daily tasks to help increase activity levels. In addition, tracking longitudinal changes in home usage could provide insights into potential cognitive, emotional, or functional declines.

### Challenges in Data Interpretation and Clinical Context

While HSH sensor data visualizations provided valuable insights, nurses faced several challenges in interpretation, particularly due to missing medical history and unclear reference points. Unclear sleep patterns made it difficult to determine whether periods of inactivity represented true sleep or simple rest without movement. One nurse noted, “Decreased sensors when asleep, with no clinical data hard to draw on inferences.” Assessing walking speed was another difficulty, as standardized clinical benchmarks were lacking. One participant questioned, “Uncertain of the difference in pace as defined by the seconds?” In addition, some nurses struggled to interpret erratic activity trends, as short bursts of movement occurred without an obvious explanation. One nurse stated, “The graphs do not show any patterns that would identify any health status change in the patient.” Finally, the lack of medical history made it challenging to draw conclusions about sleep, mobility, and bathroom usage patterns.

#### Clinical Implications

Nurses highlighted the need for better integration of HSH sensor data with medical records and clinical notes to improve interpretation accuracy. In addition, they suggested that more intuitive visualizations and standardized reference values for sleep, activity, and mobility metrics could enhance clinical decision-making.

In summary, nurses used HSH sensor data visualizations to evaluate Case 3’s sleep, mobility, and home engagement patterns, identifying frequent nighttime awakenings, walking speed variability, and reduced engagement in daily activities. However, challenges in data interpretation—including unclear sleep metrics, lack of medical history, and difficulties with mobility data interpretation—limited the ability to make definitive clinical judgments. These findings underscore the importance of improved contextualization, enhanced data visualization, and the integration of HSH sensor data visualizations in conducting assessments with clinical evaluations to support the use of smart home technology in patient care. Across all cases, data interpretation challenges limited clinical decision-making. Nurses encountered ambiguities in sleep data, questioning whether HSH sensor inactivity truly reflected sleep. Similarly, walking speed fluctuations were difficult to assess due to the lack of standardized clinical reference values.

The absence of medical history in some cases further hindered interpretation, as HSH sensor data alone lacked sufficient context to support definitive clinical conclusions. These findings highlight the need for better integration of HSH sensor data with medical records, improved data visualization techniques, and clear clinical benchmarks to enhance usability for health care professionals.

### Survey Results

A total of 14 nurse-participants were included in this study, each of whom evaluated all 3 graph types (bar, line, and pie). The 11-item (Table 1), 7-point Likert scale questionnaire provided preliminary insights into participants’ perceptions of HSH sensor data visualizations. Given that each participant responded to all 11 items for each of the 3 graph types, this resulted in 154 total responses per graph type (14 participants×11 items), yielding a dataset of 462 responses across all graph types. There was no missing data, and each response was complete, ensuring data integrity. Descriptive analysis revealed differences in perceived usability and clinical relevance across the 3 visualization types (Table 2). Bar graphs and line graphs were rated the most favorably, with participants generally agreeing that they were easy to understand and clinically useful (bar: mean 3.64‐4.57, SD 1.49‐2.22; line: mean 3.93‐4.86, SD 1.55‐2.28). In contrast, pie charts were generally rated less favorably, with more neutral to somewhat disagree responses (mean 4.14‐5.29, SD 2.10‐2.44), particularly for ease of finding information and overall clarity. The lowest-rated item for pie charts was “The organization of the visual data was clear” (mean 5.29, SD 2.23), indicating potential difficulties in interpreting structured information. These findings suggest that bar and line graphs are more effective for clinical decision-making, whereas pie charts may require design improvements to enhance interpretability and usability.

**Table 2.** Results of survey questions (N=14).

Nurses Feedback	Bar, mean (SD)	Line, mean (SD)	Pie, mean (SD)
“Overall, I am satisfied with how easy it is to understand the visualization of the data”	4.07 (2.02)	4.36 (1.74)	4.86 (2.38)
“I was able to efficiently interpret the visual data”	4.07 (1.94)	4.43 (1.55)	4.57 (2.44)
“I felt comfortable interpreting this visualization”	3.79 (2.22)	4.14 (2.28)	4.93 (2.2)
“I believe I could become productive quickly using this visualization”	4.07 (2.16)	4.21 (2.08)	4.71 (2.3)
“It was easy to find the information I needed in this visualization”	4.14 (1.96)	4.43 (1.65)	5.14 (2.21)
“The information provided by the visualization was easy to understand”	4 (2.18)	4.21 (1.72)	5 (2.15)
“The organization of the visual data was clear”	4.57 (2.03)	4.86 (1.83)	5.29 (2.23)
“The visualization contains all of the information I would expect it to have”	3.71 (1.49)	3.93 (2.02)	4.14 (2.32)
“The visual data is presented in a clinically meaningfully way”	4 (1.88)	4.36 (1.91)	4.64 (2.21)
“I am confident that visualizing data in this format would allow me to identify clinically meaningful change in health status”	3.64 (1.82)	4 (2.11)	4.14 (2.25)
“Overall, I am satisfied with this visualization”	3.86 (1.83	4.07 (1.82)	4.43 (2.1)

The Friedman test was conducted to determine whether there were significant differences in participants’ ratings of the 3 visualization types. Each participant’s individual ratings across all 11 questions for the 3 visualization types (bar, line, and pie) were included in the test to account for within-subject variability. The results indicated a statistically significant difference across the 3 visualization types (*χ*²_2_=17.1; *P*<.001), confirming that participants rated bar, line, and pie charts differently in terms of usability and clinical relevance. Post-hoc Wilcoxon signed-rank tests were conducted to determine which visualization types differed significantly in participant ratings. The results indicated that there was no significant difference between bar and line graphs (W=2857; *P*=.10), suggesting that both were perceived similarly in terms of usability and clinical relevance. However, pie charts were rated significantly lower than both bar graphs (W=1437; *P*<.001) and line graphs (W=2678.5; *P*=.008), confirming that participants found pie charts less useful for clinical decision-making. These findings support the qualitative feedback that bar and line graphs were more effective in conveying HSH sensor data, whereas pie charts presented challenges in clarity and interpretation.

### Integration of Content Analysis and Survey Findings

The findings from the 3 cases illustrate both the potential value and challenges of using HSH sensor data visualizations to assess sleep, mobility, and home engagement in older adults. Across all cases, nurses were able to identify key patterns, such as frequent nighttime awakenings, fluctuations in mobility, and variable home engagement, demonstrating how smart home technology can provide real-time insights into health status and functional changes. However, common challenges in data interpretation—including unclear sleep metrics, difficulties distinguishing true rest from inactivity, lack of clinical context, and limitations in mobility assessments—highlight the need for improved contextualization and integration of HSH sensor data with clinical evaluations.

The survey results broadly confirm the qualitative findings from the inductive content analysis, particularly in relation to the challenges nurses faced when interpreting HSH sensor data visualizations and their preferences for certain visual formats. However, the extent of this alignment varies across specific themes. The survey results confirm several key findings from the qualitative analysis, particularly in relation to challenges in interpreting smart home data. The Friedman test indicated a statistically significant difference in how nurses rated different graph types (*χ*²_2_=17.1, *P*<.001), with pie charts rated significantly lower than both bar and line graphs (*P*<.001 and *P*=.008 respectively). This supports nurses’ qualitative feedback that pie charts were less useful and harder to interpret for clinical decision-making. Nurses explicitly stated that line graphs and trend analyses were more useful, whereas pie charts lacked sufficient information, as highlighted in statements such as: *“*The pie chart didn’t provide much information*.”*

Further, the survey results confirmed difficulties in interpreting sleep and mobility data, with *“*The organization of the visual data was clear*”* receiving the lowest rating for pie charts (mean 5.29, SD 2.23). This aligns with qualitative concerns that HSH sensor data, such as sleep and mobility data, were difficult to interpret without additional clinical context. Nurses struggled to differentiate true sleep from inactivity and had difficulty interpreting walking speed metrics, questioning whether variability was due to fatigue, pain, or another factor. Statements, such as: *“*I do not know how to interpret walking speed. What is the unit of measure?*”* directly reflects the survey results, where participants rated clarity and interpretability lowest for pie charts. In addition, the survey results confirmed that both bar and line graphs were rated similarly in terms of usability and clinical relevance, with no significant difference between the two. This supports the qualitative finding that both formats were generally effective for clinical interpretation. Nurses preferred visualizations that allowed them to track trends over time, reinforcing the survey finding that bar and line graphs provided more clinically meaningful insights than pie charts. This was further validated by qualitative statements such as: *“*I used the graph charts and the links to the sensor data. The pie chart didn’t provide much information*.”* These findings collectively suggest that bar and line graphs are more effective for clinical decision-making, while pie charts may not provide sufficient clarity or actionable insights in this context.

## Discussion

### Principal Findings

The integration of smart home technologies in health care presents significant opportunities for enhancing the remote monitoring and management of older adults with chronic diseases. However, the effectiveness of these technologies is closely tied to the design of data visualizations and the proficiency of nurses in interpreting and acting upon this data.

### Co-Designing Health-Smart Home Data Visualizations With Nurses

Involving nurses in the co-design of health-smart home data visualizations is crucial. Nurses play a pivotal role in patient care and are increasingly engaged in remote health monitoring. Their insights can ensure that data visualizations are intuitive, relevant, and actionable [52]. By collaborating with nurses in the design process, visualizations can be tailored to meet clinical needs, thereby enhancing the quality of remote monitoring and patient outcomes [53]. This collaboration enhances the usability of tools and reduces cognitive load through features like color-coded alerts. It ensures that data are presented in ways that not only provide information but also support holistic knowledge of the older adult and their life contexts. Such knowledge is needed for rapid clinical decision-making in an era of precision medicine, where emphasis is placed on patients’ autonomy and right to self-determination.

Furthermore, as patient advocates, nurses can shape visualizations to facilitate communication with older adults and their families, translating complex HSH sensor data into meaningful, actionable insights for themselves. Addressing challenges like data overload or technical skepticism through co-design can also foster trust in these systems, ultimately enhancing adoption and positive end-user experiences.

### Enhancing Nurses' Proficiency in Remote Monitoring

The efficacy of smart home systems in detecting health changes is significantly influenced by nurses’ proficiency in using data dashboards, alongside access to clinical history and in-person collected data. A study examining nurses’ practices in remote monitoring of patients with chronic diseases found that nurses use data in the form of graphs, alerts, questionnaires, and messages to personalize care [54]. This underscores the necessity for comprehensive training programs that equip nurses with the skills to interpret complex HSH sensor data and integrate it with traditional clinical assessments and medical history. In addition to the design of visualization tools, nurses’ own competencies in data literacy and numerical literacy are critical factors influencing interpretation. Studies highlight that variability in nurses’ confidence with quantitative data can affect how effectively they identify patterns or assess clinical significance [29,38]. Integrating targeted education on data and numerical literacy into training programs may therefore enhance the safe and effective use of HSH sensor data. Furthermore, the development and implementation of nurse-based remote patient monitoring programs have demonstrated that dashboards available through software platforms allow RNs to monitor patients effectively, viewing and responding to real-time alerts when data falls outside predefined limits. This integration of technology into nursing practice not only enhances patient care but also supports nurses in making informed decisions based on a combination of real-time data and clinical expertise [55].

### New Types of Evidence for Evidence-Based Practice

Introducing nurses to data derived from unobtrusive, continuous ambient sensing is important to growing their knowledge about how to use this new type of data as evidence for evidence-based decision-making. Nurses and other health care team members are currently not trained to use this type of data. This will result in the need for training to increase comfort with relying on the data and associated visuals (graphs and charts). We posit that obtaining knowledge and comfort with information provided by smart homes will require similar changes to thinking about data as the introduction of smart infusion pumps, automated medication dispensing machines, and other information derived from remote patient monitoring equipment. Embracing a growth mindset as new forms of data emerge, including information outputs from devices featuring artificial intelligence, will position nurses for the future of health care.

### Nurses as Future Data Brokers

Given that artificial intelligence capabilities will continue to rapidly change how and when we receive information about the health of patients, and given nurses are currently the information hub for most complex cases (eg, case managers), they are well-positioned to be future health care information data brokers. Nurses already use scientific principles, mathematical concepts, and cutting-edge technology featuring artificial intelligence to assess, diagnose, and plan patient care [56]. As nurses become experts in new and more sophisticated technologies, they will likely lead their health care teams in translating HSH sensor data into knowledge that reliably informs care decisions and interventions.

### Limitations

The small sample size and characteristics restrict generalizability of the findings, and participants’ perspectives may not reflect the experiences of nurses in different clinical settings. In addition, the study relied on self-reported interpretations, which introduces the potential for bias. No participants had prior experience working with smart home data; however, all were experienced nurses with at least 5 years of clinical practice. In addition, the scenarios chosen for this study were vetted by a nurse scientist (RF) having experience working with HSH sensor data and creating visualizations. Scenarios were purposively chosen containing data about health events that subjectively represented easy, medium, and hard-to-interpret data. No scenarios were presented that were deemed impossible to interpret. Future research should examine how nurses with varying levels of data literacy and technology exposure engage with HSH sensor data visualizations, incorporate objective usability assessments and comparative studies to strengthen the reliability of findings, and should explore how training in HSH data literacy and digital health tools impacts nurses’ ability to use HSH sensor data effectively.

### Conclusion

This study provides new insights into nurses’ interpretation of health-smart home-generated data, revealing key challenges in sleep, mobility, and activity trend analysis. Findings highlight the need for improved visualization techniques, clinician training, and standardized benchmarks to optimize clinical decision-making in home care settings using HSH sensor data visualizations.

## Abbreviations

ADL: Activities of daily living
ECG: Electrocardiogram
EU: European Union
HSH: Health-smart home
IRB: Institutional Review Board
PROMS: Patient-reported outcome measures
PSSUQ: Post-Study System Usability Questionnaire
RN: Registered Nurse
